# Supplementary material for: PRO-C3 is a predictor of clinical outcomes in distinct cohorts of patients with advanced liver disease
Source: JHEP Rep. 2023 Mar 28;5(6):100743. doi: 10.1016/j.jhepr.2023.100743 (PMC10240276; doi:10.1016/j.jhepr.2023.100743)
Supplement: Multimedia component 1 [file mmc1.pdf]

PRO-C3 is a predictor of clinical outcomes in distinct cohorts of patients with advanced liver disease

Mette J Nielsen[1], Grace E Dolman[2], Rebecca Harris[2], Peder Frederiksen[1], Jane Chalmers[2], Jane Grove[2,5], William L Irving[2,4], Morten A Karsdal[1], Keyur Patel[3], Diana Julie Leeming[1], Indra Neil Guha[2,5]

Table of contents

Fig. S1……………………………………………………………………………………1

Fig. S2……………………………………………………………………………………2

Fig. S1

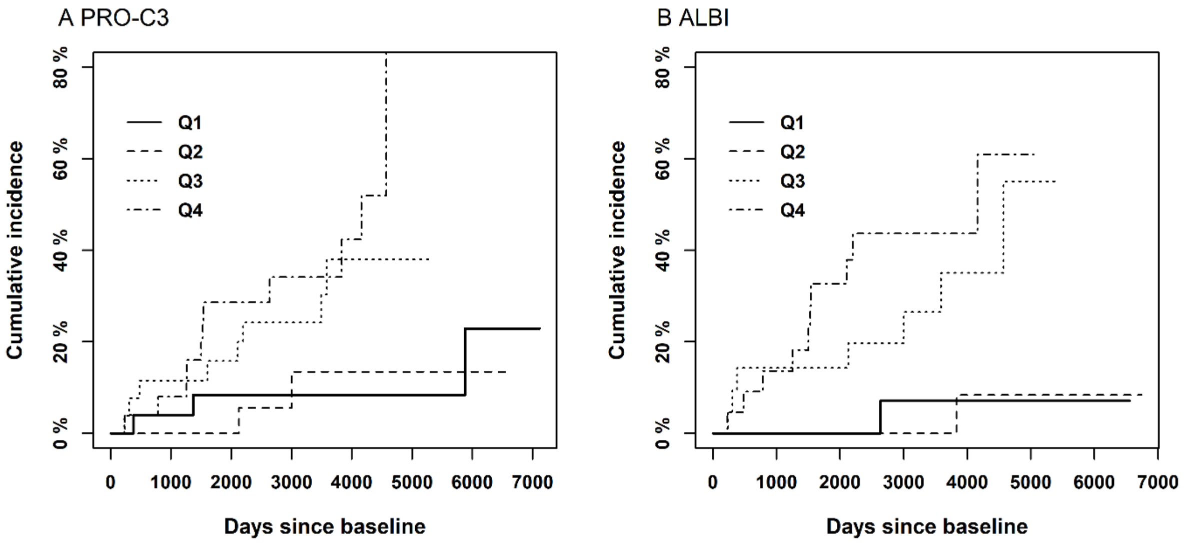
A PRO-C3
B ALBI
Cumulative incidence
0 %
20 %
40 %
60 %
80 %
Q1
Q2
Q3
Q4
0
1000
2000
3000
4000
5000
6000
7000
Days since baseline

Fig. S2

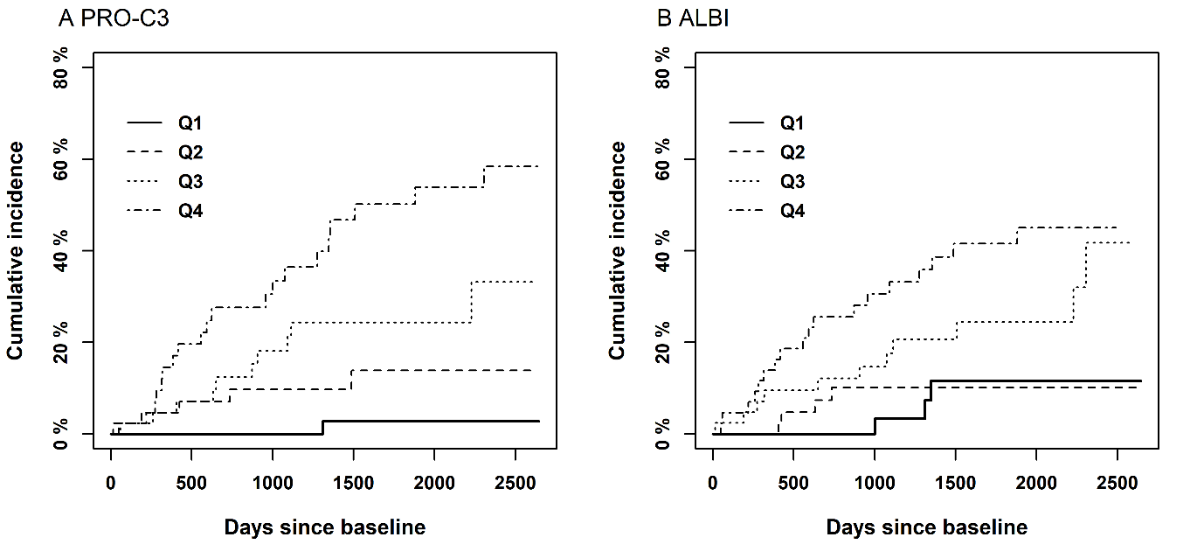
A PRO-C3
B ALBI
Cumulative incidence
0 %
20 %
40 %
60 %
80 %
Q1
Q2
Q3
Q4
0
500
1000
1500
2000
2500
Days since baseline
