# Supplementary material for: PRO-C3 is a predictor of clinical outcomes in distinct cohorts of patients with advanced liver disease
Source: JHEP Rep. 2023 Mar 28;5(6):100743. doi: 10.1016/j.jhepr.2023.100743 (PMC10240276; doi:10.1016/j.jhepr.2023.100743)
Supplement: Multimedia component 4 [file mmc4.pdf]

PRO-C3 is a predictor of clinical outcomes in distinct cohorts of patients with advanced liver disease

Mette J. Nielsen,[1] Grace E. Dolman,[2] Rebecca Harris,[2] Peder Frederiksen,[1] Jane Chalmers,[2] Jane I. Grove,[2,3] William L. Irving,[2,4] Morten A. Karsdal,[1] Keyur Patel,[5] Diana Julie Leeming,[1] Indra Neil Guha[2,3,*]

[1]Nordic Bioscience, Herlev, Denmark; [2]National Institute for Health Research (NIHR) Nottingham Biomedical Research Centre, Nottingham University Hospitals NHS Trust and University of Nottingham, Nottingham, UK; [3]Nottingham Digestive Diseases Centre, School of Medicine, University of Nottingham, Nottingham, UK; [4]School of Life Sciences, University of Nottingham, Nottingham, UK; [5]Division of Gastroenterology and Hepatology, University of Toronto Health Network, Toronto, ON, Canada

JHEP Reports **2023.** https://doi.org/10.1016/j.jhepr.2023.100743

Background & Aims: Fibroblast activity is a key feature of fibrosis progression and organ function loss, leading to liver-related complications and mortality. The fibrogenesis marker, PRO-C3, has been shown to have prognostic significance in relation to fibrosis progression and as a treatment efficacy marker. We investigated whether PRO-C3 was prognostic for clinical outcome and mortality in two distinct cohorts of compensated cirrhosis.
Methods: Cohort 1 was a rapid fibrosis progression cohort including 104 patients with HCV and biopsy-proven Ishak fibrosis stage ≥3 without prior clinical events. Cohort 2 was a prospective cohort including 172 patients with compensated cirrhosis of mixed aetiology. Patients were assessed for clinical outcomes. PRO-C3 was assessed in serum at baseline in cohorts 1 and 2, and compared with model for end-stage liver disease and albumin–bilirubin (ALBI) scores.
Results: In cohort 1, a 2-fold increase in PRO-C3 was associated with 2.7-fold increased hazard of liver-related events (95% CI 1.6–4.6), whereas a one unit increase in ALBI score was associated with a 6.5-fold increased hazard (95% CI 2.9–14.6). In cohort 2, a 2-fold increase in PRO-C3 was associated with a 2.7-fold increased hazard (95% CI 1.8–3.9), whereas a one unit increase in ALBI score was associated with a 6.3-fold increased hazard (95% CI 3.0–13.2). A multivariable Cox regression analysis identified PRO-C3 and ALBI as being independently associated with the hazard of liver-related outcomes.
Conclusions: PRO-C3 and ALBI were independent prognostic factors for predicting liver-related clinical outcomes. Understanding the dynamic range of PRO-C3 might enhance its use for both drug development and clinical practice.
Impact and Implications: We tested novel proteins of liver scarring (PRO-C3) in two groups of liver patients with advanced disease to see if they could predict clinical events. We found that this marker and an established test called ALBI were both independently associated with future liver-related clinical outcomes.
© 2023 The Authors. Published by Elsevier B.V. on behalf of European Association for the Study of the Liver (EASL). This is an open access article under the CC BY license (http://creativecommons.org/licenses/by/4.0/).

Introduction

Evolution of fibrosis to cirrhosis represents the initiation and perpetuation of liver injury resulting in extracellular matrix (ECM) deposition and architectural disturbance. The transition from compensated to decompensated cirrhosis heralds a significant change in prognosis. The ability to predict which patients are at greatest risk of decompensation is important from three different perspectives. Firstly, it provides patients with an informed and objective trajectory of their disease. Secondly, it allows practitioners to monitor patients and offer timely intervention. Finally, it provides healthcare systems with tools to stratify risk and enable proportionate resources to be directed at those at greatest risk of a clinical outcome. Prognostic tools such as the model for end-stage liver disease (MELD) score offer excellent performance once significant synthetic failure has occurred but have limitations, which have been well described.[1–3] Recently, simple scores such as albumin–bilirubin (ALBI), extensively validated in the context of hepatocellular carcinoma (HCC),[4] have also been shown to stratify compensated cirrhosis, with no overt synthetic failure, into categorical prognostic groups, but there remains scope to improve upon this.[5,6]

In active hepatic fibrogenesis, accelerated ECM remodelling leads to 6-fold increased deposition of ECM components in the liver, especially types I, III, and IV collagens.[7] Fibroblast activity is a key feature of driving fibrosis progression and organ function loss, and the fibroblast is the principal promotor of ECM accumulation in fibrotic disorders, including liver, cardiovascular, and chronic kidney diseases, as well as cancer.[8] In addition, fibrosis stage is the main driver of mortality.[9] Proteases such as matrix metalloproteinases are responsible for remodelling of the hepatic ECM during progression of fibrosis, generating uniquely

Keywords: Cirrhosis; Biomarker; Outcome; Extracellular matrix.
Received 16 November 2022; received in revised form 24 February 2023; accepted 15 March 2023; available online 28 March 2023
* Corresponding author. Address: NIHR Nottingham Biomedical Research Centre, Nottingham University Hospitals NHS Trust, University of Nottingham, Derby Road, Nottingham, UK. Tel.: +44-115-823-1162.
E-mail address: Neil.guha@nottingham.ac.uk (I.N. Guha).

modified fragments, neo-epitopes, which are released into the circulation[10,11] and may be used as surrogate measures of ECM remodelling and prognostication. Serum levels of these markers not only reflect disease activity in the liver but are also associated with the severity of fibrosis and portal hypertension in experimental and human settings[12–15] The evidence of diagnostic and prognostic performances has largely been based on studies showing accuracy against conventional measures of liver fibrosis defined by invasive histological measurement. This is a surrogate measurement, and defining the relationship of ECM remodelling markers directly with clinical outcomes is of greater importance.

Our primary objective was to assess whether fibrosis activity assessed as active fibrogenesis, represented by PRO-C3 (formation of type III collagen)[16] provides prognostic utility in two distinct cohorts of compensated cirrhosis. The first, a retrospective cohort, included assessment against histological outcomes, and the second, a prospective cohort, representing contemporaneous care, used hard clinical outcomes.

Patients and methods

Cohort 1: Trent retrospective study

This was a retrospectively identified study cohort derived from the Trent Study of Patients with Hepatitis C Virus Infection, as described previously.[17,18] This cohort was established in 1991 to study the epidemiology and natural history of hepatitis C infection in a defined administrative health region of the UK with a population of over 5 million people. The study prospectively enrolled patients with chronic hepatitis C (CHC) who attended routine clinical appointments and collected health-related information, demographics, and biospecimens for future research use.

Ethics were formally reviewed and approved by the regional committee (Northern and Yorkshire MREC98/3/55). Patients were selected from a single centre within the Trent HCV cohort between 1993 and 2010 based on criteria that included the following: (i) liver biopsy before antiviral therapy; (ii) HCV RNA positive at biopsy; (iii) Ishak stage ≥3, as determined by an independent tertiary centre histopathologist blinded to other study or clinical information; and (iv) no clinical outcome before the liver biopsy (as defined below).

Medical records were reviewed to collect data pertaining to clinically significant outcomes, defined as the first event recorded of the following: (i) ascites requiring treatment; (ii) variceal bleeding requiring endoscopic therapy; (iii) overt hepatic encephalopathy (grade 2, 3, or 4 West Haven classification); (iv) HCC (defined by EASL criteria); (v) orthotopic liver transplantation; or (vi) liver-related death. If there were multiple events, only the first chronological event was used for analysis. To capture clinical events in patients who had moved away from the original enrolling centre, these study participants were identified in the National Health Service Central Register to obtain details pertaining to date and cause of death and cancer registration data.

Patients who did not reach a clinical outcome during the follow-up period were censored at the time of either (i) last seen in clinic without evidence of liver-related clinical outcome or (ii) non-liver-related death. All patients had sera obtained within 6 months of the liver biopsy and stored at -80 °C until analysis. Patients with CHC were eligible to receive pegylated interferon and ribavirin as standard-of-care therapy at the time (before the direct-acting antiviral [DAA] era).

Cohort 2: compensated cirrhosis cohort (prospective study)

Patients were consecutively recruited from the Nottingham Compensated Cirrhosis Cohort study (3CN). The 3CN study is a prospective, longitudinal study initiated in 2010 focusing on the study of early compensated liver cirrhosis. The study was approved by an NHS ethics committee, and standard regulatory requirements obtained (10/H0403/10). Inclusion criteria were patients between the ages of 18 and 75 years and an established diagnosis of cirrhosis obtained by at least one of the following criteria:

- confirmation of cirrhosis by histology, imaging, or non-bleeding gastroesophageal varices on endoscopy; and
- clinical evidence of cirrhosis with thrombocytopaenia (platelet count <150,000) and a validated non-invasive liver fibrosis test (transient elastography >15 kPa).[19]

Exclusion criteria included the following:

- presence of HCC at baseline,
- portal or splenic vein thrombosis,
- clinical or radiological ascites at baseline visit,
- history of variceal haemorrhage,
- any previous episode of clinical encephalopathy,
- non-cirrhotic portal hypertension, and
- history of organ transplant or end-stage renal disease requiring dialysis.

The primary outcome was a liver-related clinical outcome. This was defined using the clinical parameters of the following: (i) the first episode of ascites (as defined by confirmation with ultrasonography and requiring treatment with diuretics or paracentesis); (ii) initial variceal bleed (defined by requiring endoscopic intervention); (iii) the initial episode of encephalopathy, assessed by an experienced clinician and defined by grade 3/4 West Haven classification; (iv) HCC (as defined by EASL criteria[20,21]), or (v) liver-related death. If there were multiple events, only the first chronological event was used for analysis.

Patients were followed up at 6-monthly visit appointments and assessed for liver-related clinical outcomes. At the end of the study, all patients were assessed for clinical outcomes using hospital records and by contacting primary care physicians directly in those failing to attend secondary care. Patients were censored if they underwent liver transplantation, died, or were at the end of the follow-up period (1 December 2017).

Quantification of ECM-related biochemical markers

De-identified serum samples were analysed using competitive ELISAs for the assessment of type III collagen formation (PRO-C3)[16] (Nordic Bioscience, Denmark).

PRO-C3 detects a fragment of the N-terminal pro-peptide of type III procollagen exclusively derived from ADAMTS-2 cleavage of the N-terminal propeptide of type III collagen during maturation and deposition, that is, during tissue formation and not during degradation, in contrast to the classical PIIINP (type III procollagen peptide) assay, which assesses an internal fragment that may be released during both type III collagen formation and degradation.[16]

Table 1. Baseline demographics of the Trent cohort.

	All patients (n = 104)
Sex (male), n (%)	74 (71)
Age (years)	46.0 (40.0–52.5)
BMI (kg/m^2)*	26.6 (22.8–29.0)
HCV genotype†, n (%)	38 (39)
1	57 (58)
2/3	3 (3)
4/5	
Obtained SVR, n (%)	39 (38)
Ishak stage, n (%)	42 (41)
3	21 (20)
4	20 (19)
5	21 (20)
6	
Heavy alcohol abuse (>150 U/week), n (%)	5 (5)
Biochemistry	
PRO-C3 (ng/ml)	33.2 (17.8–55.0)
ALT	105.0 (72.0–172.0)
ALBI	-2.7 (-2.9 to -2.4)
Liver-related outcomes, n (%)	24 (23)
Follow-up (years), mean (range)	7.9 (0.13, 19.5)

ALBI, albumin–bilirubin; ALT, alanine aminotransferase; SVR, sustained virologic response.
* BMI, n = 89.
† Genotype, n = 98.

Statistical analysis

Differences between categorical variables were analysed using Fisher's exact test. Comparison between biomarker levels were performed using the Kruskal–Wallis test. Differences between patients with events compared with patients without were compared using the Mann–Whitney test. Non-parametric Aalen–Johansen estimates were used to evaluate the association between biomarker levels and the absolute risk of clinical outcomes, treating non-liver-related deaths as competing risks. Median baseline levels or quartiles were used to categorise the biomarker levels into low or high. In addition to presenting performance of the markers using non-parametric estimates of the absolute risk of liver-related outcomes within quartiles of PRO-C3 or ALBI, we also examined performance by using the markers as continuous variables for describing the hazard of liver-related outcomes using Cox regression. In the 3CN cohort, a multivariable analysis was performed using PRO-C3, age, sex, BMI, alanine aminotransferase (ALT), and the markers of prognosis MELD and ALBI scores.[5,22] To linearise its effect on the hazard, PRO-C3 was log-transformed in the regression analyses. A nominal 5% level of significance was used throughout the analyses. R version 4.0.3 (R Core Team, 2020, R Foundation for Statistical Computing, Vienna, Austria) was used for all statistical calculations.

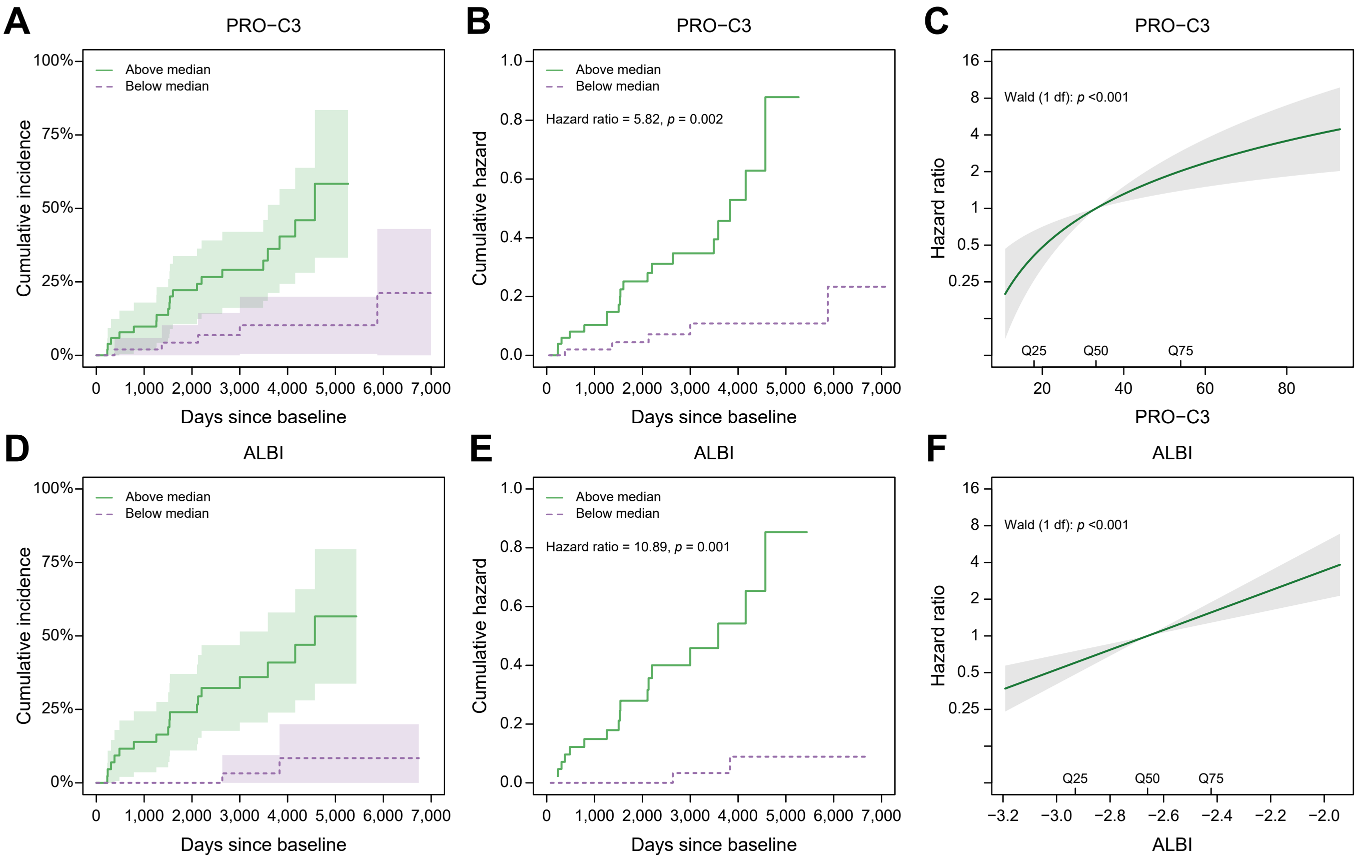

Fig. 1. Association between increasing biomarker level and risk of developing a clinical event for PRO-C3 and ALBI in the Trent cohort. Aalen–Johansen estimates of the cumulative incidence of liver-related outcome stratified by (A) baseline median PRO-C3 levels and (D) baseline median ALBI in the Trent cohort. Shaded areas represent 95% CIs. Nelson–Aalen estimates of the cumulative hazard of liver-related outcome in the Trent cohort for (B) PRO-C3 baseline levels and (E) ALBI baseline levels below and above the median. Hazard ratio for liver-related outcome according to increasing biomarker level for (C) PRO-C3 and (F) ALBI estimated by Cox regression in the Trent cohort. The baseline hazard corresponds to the median level of each biomarker. ALBI, albumin–bilirubin.

Table 2. Baseline demographics of the 3CN cohort.

	All patients (n = 172)
Sex (male), n (%)	110 (64)
Age (years)	61.0 (54.5–66.0)
BMI (kg/m^2)	30.0 (26.7–34.2)
Aetiology, n (%)	56 (33)
NAFLD	69 (40)
ALD	26 (15)
HBV/HCV	20 (12)
Other	
MELD	7.5 (6.4–8.5)
Biochemistry	
PRO-C3 (ng/ml)	21.0 (13.3–34.3)
ALT	34.0 (24.3–52.0)
ALBI	-2.5 (-2.8 to -2.2)
Liver-related outcomes, n (%)	36 (21)
Follow-up (years), mean (range)	3.9 (0.04, 7.3)

3CN, Compensated Cirrhosis Cohort in Nottingham; ALBI, albumin–bilirubin; ALD, alcoholic liver disease; ALT, alanine aminotransferase; MELD, model for end-stage liver disease; NAFLD, non-alcoholic fatty liver disease.

Results

Cohort 1: the retrospective Trent study

In total, 104 patients with CHC were included in this study. Demographics, including genotype, alcohol intake, BMI, and baseline fibrosis stage, are shown in Table 1. Median age was 46.0 years, 74% of the patients were male, and 41/104 (39%) had Ishak stage 5–6. Thirty-nine (39%) patients achieved sustained virologic response (SVR) during the follow-up period (median 7.7 years). Median ALBI score was -2.7, reflecting low mortality risk,[23] and median PRO-C3 was 33.2 ng/ml. Overall, 24/104 (23%) had a liver-related outcome, including two post-SVR patients. The outcomes included ascites (n = 10), HCC (n = 12), and liver-related death (n = 2).

We investigated the association between increasing biomarker level and risk of developing a clinical event for PRO-C3 and ALBI. When patients were stratified into baseline PRO-C3 levels above or below the median level, patients with high PRO-C3 levels had 58% (95% CI 33–83%) risk of liver-related events compared with 21% (95% CI 0–43%) risk for patients with low PRO-C3 (Fig. 1A) before the end of follow-up. The hazard of liver-related outcomes was 5.8-fold higher among patients with PRO-C3 levels above the median, compared with patients with PRO-C3 levels below the median (hazard ratio [HR] 5.8, 95% CI 2.0–17.3, p = 0.002) (Fig. 1B). Using PRO-C3 on a continuous scale showed that PRO-C3 was associated with the hazard of liver-related events (p <0.001) (Fig. 1C). Every 2-fold increase in the biomarker level was associated with a 2.7-fold (95% CI 1.6–4.6) increase in the hazard of events. Likewise,

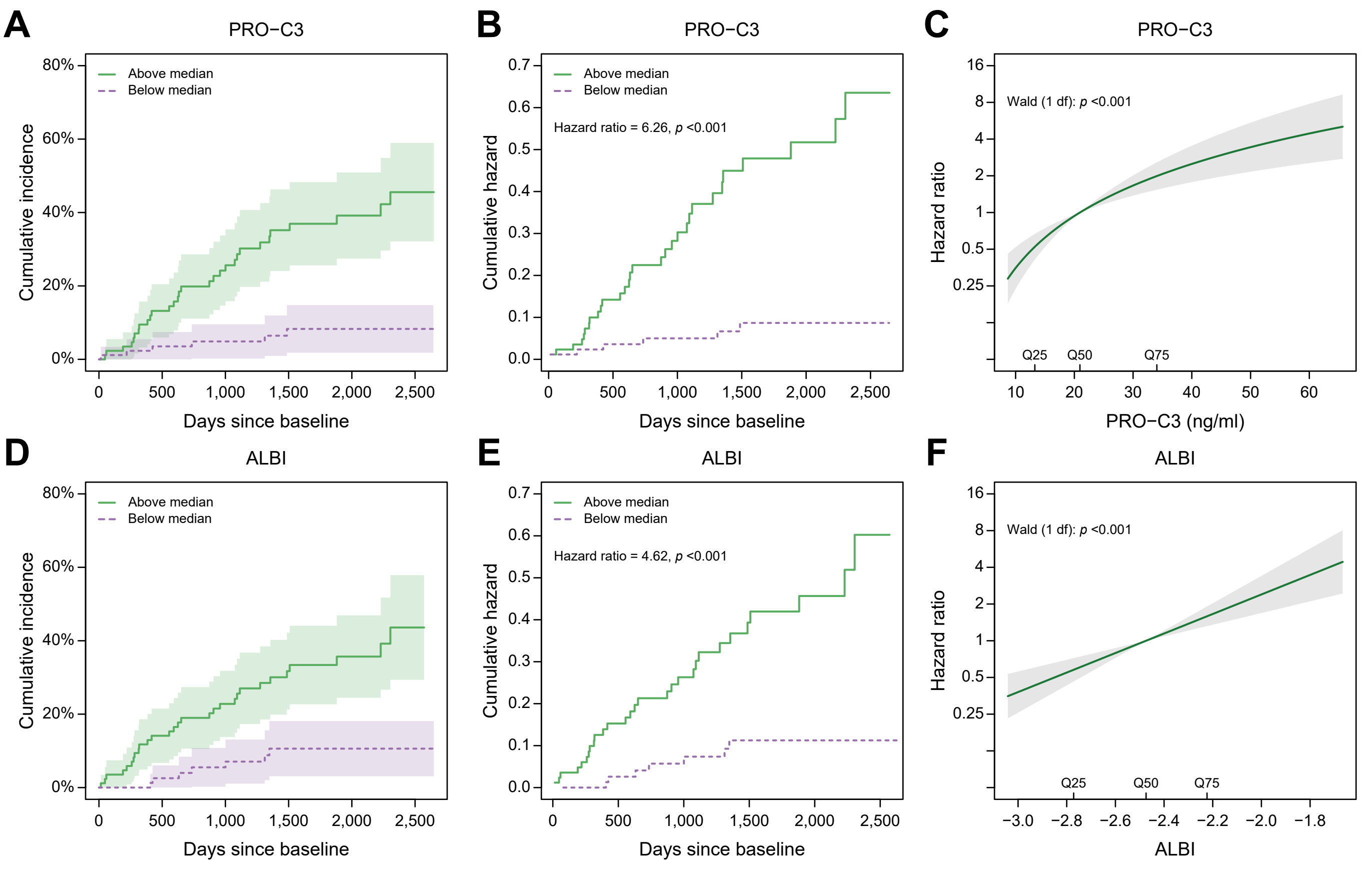

Fig. 2. Association between increasing biomarker level and risk of developing a clinical event for PRO-C3 and ALBI in the 3CN cohort. Aalen–Johansen estimates of the cumulative incidence of liver-related outcome stratified by (A) baseline median PRO-C3 levels and (D) baseline median ALBI in the 3CN cohort. Shaded areas represent 95% CIs. Nelson–Aalen estimates of the cumulative hazard of liver-related outcome in the 3CN cohort for (B) PRO-C3 baseline levels and (E) ALBI baseline levels below and above the median. Hazard ratio for liver-related outcome according to increasing biomarker level for (C) PRO-C3 and (F) ALBI estimated by Cox regression in the 3CN cohort. The baseline hazard corresponds to the median level of each biomarker. 3CN, Compensated Cirrhosis Cohort in Nottingham; ALBI, albumin–bilirubin.

Table 3. Multivariable Cox regression analyses of liver-related outcomes in the 3CN cohort.

	Hazard ratio [95% CI]	*p*
PRO-C3*	2.24 [1.43, 3.50]	<0.001
Age	0.99 [0.96, 1.03]	0.728
Male:female	0.84 [0.37, 1.88]	0.664
BMI	0.98 [0.92, 1.05]	0.590
MELD	1.00 [0.85, 1.19]	0.983
ALBI	3.24 [1.28, 8.19]	0.013
ALT	1.00 [0.99, 1.01]	0.980

* Per 2-fold increase. 3CN, Compensated Cirrhosis Cohort in Nottingham; ALBI, albumin–bilirubin; ALT, alanine aminotransferase.

when patients were stratified into ALBI score above or below the median at baseline, patients with high ALBI had 57% (95% CI 34–80%) risk of liver-related events compared with 8% (95% CI 0–20%) risk for patients with low ALBI (Fig. 1D) before the end of follow-up. Furthermore, the hazard of liver-related outcomes was 11-fold higher among patients with ALBI scores above the median, compared with patients with ALBI scores below the median (HR 11.0, 95% CI 2.5–47.2, p = 0.001) (Fig. 1E). Using ALBI on a continuous scale showed that ALBI was associated with the hazard of liver-related events (p <0.001) (Fig. 1F). A one unit increase in the score was associated with a 6.5-fold (95% CI 2.9–14.6) increase in the hazard of events.

The Cox regression analyses indicate a large variation in the hazard of events from the lowest levels of PRO-C3 or the ALBI score to the highest levels. When stratifying the patients according to baseline quartiles, we observe a very low absolute risk of events in Q1 and a relatively high risk in Q4 (Fig. S1).

Cohort 2: the prospective 3CN study

In total, 172 patients were included in the 3CN cohort. Demographics, including aetiology, BMI, and baseline disease severity, are shown in Table 2. Median age was 61 years, and 64% were male. Alcoholic (40%) and non-alcoholic fatty liver disease (33%) were the major aetiologies for advanced liver disease. No differences in baseline PRO-C3 levels were found between aetiologies, and thus, results were pooled for analysis. Median MELD score was 7.5, and median ALBI score was -2.5, reflecting compensated disease with intermediate mortality risk. Median PRO-C3 was 21 ng/ml. Moreover, 36/174 (21%) patients of this cohort reached a liver-related clinical outcome over a median of 3.8 years. There were a total of 36 liver-related outcomes, which included ascites (n = 20), hepatic encephalopathy (n = 4), variceal bleeding (n = 4), HCC (n = 5), and liver-related death (n = 3).

We investigated the association between increasing biomarker level and risk of developing a clinical event for PRO-C3 and ALBI in a similar manner to that in cohort 1. In the 3CN cohort, PRO-C3 was associated with the risk of liver-related outcomes (Fig. 2A). Patients with PRO-C3 levels above the median had 46% (95% CI 32–59%) risk of developing a liver-related outcome (Fig. 2A) before the end of follow-up. The risk among patients with PRO-C3 levels below the median was 8% (95% CI 2–15%). The hazard of liver-related outcomes was 6.1-fold higher among patients with PRO-C3 levels above the median than among patients with PRO-C3 levels below the median (HR 6.1, 95% CI 2.6–15.1, p <0.001) (Fig. 2B). Using PRO-C3 on a continuous scale showed that PRO-C3 was associated with the hazard of liver-related events (p <0.001) (Fig. 2C). Every 2-fold increase in PRO-C3 was associated with a 2.7-fold (95%CI 1.8–3.9) increase in the hazard of events.

ALBI was associated with the risk of liver-related outcomes (Fig. 2D). Before the end of follow-up, patients with ALBI scores above the median had 44% (95% CI 29–58%) risk of developing a liver-related outcome (Fig. 2D). The risk among patients with ALBI scores below the median was 11% (95% CI 3–18%). The hazard of liver-related outcomes was 4.6-fold higher among patients with ALBI scores above the median, compared with patients with ALBI scores below the median (HR 4.6, 95% CI 2.0–11.0, p <0.001) (Fig. 2E). Using ALBI on a continuous scale showed that ALBI was associated with the hazard of liver-related events (p<0.0.001), and a one unit increase in ALBI was associated with a relative increase in the hazard of events of 6.8 (95% CI 3.0–13.2) (Fig. 2F).

Similar to the findings in cohort 1, the Cox regression analyses indicate a large variation in the hazard of events from the lowest levels of PRO-C3 or the ALBI score to the highest levels. When stratifying the patients according to baseline quartiles, we observe a very low risk of events in Q1 and a relatively high risk in Q4 (Fig. S2).

Lastly, we investigated whether PRO-C3 and ALBI score were independent predictors of clinical outcomes by multivariable Cox regression analyses, showing that PRO-C3 and ALBI were independently associated with the hazard of liver-related events (Table 3).

Discussion

Our study is the first to assess and validate PRO-C3 for clinical outcomes in advanced liver disease and has revealed three principal findings. Firstly, PRO-C3 provides prognostic utility in both the historical HCV cohort and prospective 3CN cohort (median threshold showing HR of 4.3 and 6.3, respectively). Secondly, the rate of disease progression has a major influence on performance. In both cohorts, PRO-C3 at baseline was significantly different in patients that went on to develop a clinical outcome, compared with patients that remained clinically stable. Thirdly, using PRO-C3 as a continuous variable reflecting active fibrogenesis, and an evolving risk assessment beyond a dichotomised endpoint such as advanced fibrosis, highlights the added value in prognostic performance. The study also showed the excellent performance of ALBI, when used as both a categorical variable and a continuous variable in predicting clinical events in both the HCV and 3CN cohorts (median threshold HR of 11 and 4.6, respectively).

There have been several studies looking at the prognostic ability of markers of ECM remodelling.[24–27] For PRO-C3, the previous research has focused on cross-sectional performance in comparison with the gold standard of histology.[13,28–34] Assessing whether PRO-C3 may provide prognostic utility addresses an important evidence gap. Furthermore, a lowering effect of DAA therapy in patients with stage 4 fibrosis with HCV on PRO-C3 has been observed, allowing us to speculate that PRO-C3 may also be a pharmacodynamic marker.[35]

The cohorts selected provide a very different clinical context, which was the deliberate intention. The Trent HCV cohort is a historical cohort treated with pegylated interferon and ribavirin before the DAA era. The low SVR rate and high clinical outcomes provide no extrapolation to the current management of HCV. Thus, the rationale of using this cohort was not to extrapolate to the current natural history of HCV but to provide a clinical phenotype of liver fibrosis progression that allowed us to assess the relationship between baseline histology and our candidate markers in the context of prognosis. This concept has been used

by a number of biomarker studies from the Hepatitis C Antiviral Long-Term Treatment Against Cirrhosis (HALT-C) study, showing independent association of histology and non-invasive markers of fibrosis with outcomes.[36,37] The 3CN cohort represents 'real-world' aetiology. The ability of markers to perform across different aetiologies is important with the increasing recognition of co-existing drivers of chronic liver disease (*e.g.* alcohol and components of the metabolic syndrome). Our study did not reveal any difference in performance between aetiologies in the compensated cohort (data not shown); however, these subpopulations were too small to definitively answer this question. Multiple studies with PRO-C3 included have been reported for multiple liver indications, including cholestatic,[38,39] metabolic,[12,14,40] and viral diseases,[13,29] showing that the type of insult determines the level of PRO-C3, and thus indicate that fibroblast activity may differ across aetiology. The difference in active fibrogenesis between aetiologies are further demonstrated in intervention studies, where the baseline level of PRO-C3 and the underlying disease activity determine the level of which PRO-C3 can be modulated by different treatment regimens.[35,41–44] The differences in baseline PRO-C3 between the retrospective HCV cohort and the 3CN cohort are likely to be related to differing pro-inflammatory environments and fibrosis stage severity, and so this effect does need to be dissected out in further validation studies.

Understanding biomarker performance for predicting clinical outcomes would enhance our ability to manage chronic liver disease. At a community level, a low cost and widely available prognostic test, such as ALBI, is attractive as an initial population stratification strategy. This study reinforces the prognostic accuracy of ALBI, which can be calculated from readily collected routine laboratory measures and has advantages over MELD in compensated liver disease. For those with progressive disease, within the higher risk strata, more careful monitoring and follow-up will be required. Improving selection of patients who are most likely to benefit from emerging antifibrotics remains a challenge, and finding markers, such as fibrogenesis markers, is an intuitive approach for selecting and monitoring the response of patients. Using the dynamic range of a biomarker to potentially assess this change is an important insight from this study. The signal from this study is that ECM epitopes, when used within the breadth of a continuous dynamic range, offer additional information, above and beyond existing tests such as ALBI and MELD scores. Comparison against other prognostic biomarkers, including transient elastography and magnetic resonance imaging, needs to be assessed in future studies. This study did not measure the longitudinal changes in biomarker levels or changes in important lifestyle factors (*e.g.* alcohol and weight), and both of these aspects will influence long term prognosis.

In conclusion, we identified PRO-C3 as an independent prognostic factor in predicting liver-related clinical outcomes in two distinct models of chronic liver injury. Understanding the dynamic range of PRO-C3 will enhance how it is used in drug development and clinical practice.

Abbreviations

3CN, Compensated Cirrhosis Cohort in Nottingham; ALBI, albumin–bilirubin; ALD, alcoholic liver disease; ALT, alanine aminotransferase; CHC, chronic hepatitis C; DAA, direct-acting antiviral; ECM, extracellular matrix; HALT-C, Hepatitis C Antiviral Long-Term Treatment Against Cirrhosis; HCC, hepatocellular carcinoma; MELD, model for end-stage liver disease; NAFLD, non-alcoholic fatty liver disease; SVR, sustained virologic response.

Financial support

This paper presents independent research funded by the National Institute for Health Research (NIHR). The views expressed are those of the authors and not necessarily those of the NHS, the NIHR, or the Department of Health.

This was an investigator-led study and joint collaboration between the University of Nottingham and Nordic Bioscience. No direct funding was received from Nordic Bioscience in grant income or consultancy fees.

Conflicts of interest

MJN, MK, PF, and DJL are full-time employees at Nordic Bioscience. MJN, MK, and DJL hold stocks in Nordic Bioscience.

Please refer to the accompanying ICMJE disclosure forms for further details.

Authors' contributions

Collagen biomarker analysis was performed by MN, DJL, JM, PF, KP and ING. The academic authors designed the clinical study (KP, WI and ING), had full independence in obtaining the clinical data (GD, BH, JG and JC) and interpretation of the data (all authors), DJL, MN, MAK selected the extracellular matrix related biomarker strategy. MN, DJL, KP and ING drafted the initial manuscript and all authors reviewed and agreed the final manuscript.

Data availability

All data generated or analysed during this study are included in this published article (and its supplementary information files).

Supplementary data

Supplementary data to this article can be found online at https://doi.org/10.1016/j.jhepr.2023.100743.

References

[1] Cholongitas E, Papatheodoridis GV, Vangeli M, Terreni N, Patch D, Burroughs AK. Systematic review: the model for end-stage liver disease – should it replace Child–Pugh's classification for assessing prognosis in cirrhosis? Aliment Pharmacol Ther 2005;22:1079–1089.

[2] Cholongitas E, Senzolo M, Triantos C, Samonakis D, Patch D, Burroughs AK. MELD is not enough – enough of MELD? J Hepatol 2005;42:475–477.

[3] Bernardi M, Gitto S, Biselli M. The MELD score in patients awaiting liver transplant: strengths and weaknesses. J Hepatol 2011;54:1297–1306.

[4] Toyoda H, Johnson PJ. The ALBI score: from liver function in patients with HCC to a general measure of liver function. JHEP Rep 2022;4:100557.

[5] Guha IN, Harris R, Berhane S, Dillon A, Coffey L, James MW, et al. Validation of a model for identification of patients with compensated cirrhosis at high risk of decompensation. Clin Gastroenterol Hepatol 2019;17:2330–2338.e1.

[6] Hsu CY, Parikh ND, Huo TI, Tapper EB. Comparison of seven noninvasive models for predicting decompensation and hospitalization in patients with cirrhosis. Dig Dis Sci 2021;66:4508–4517.

[7] Arriazu E, Ruiz De Galarreta M, Cubero FJ, Varela-Rey M, Pé Rez De Obanos MP, Leung TM, et al. Extracellular matrix and liver disease. Antioxid Redox Signal 2014;21:1078–1097.

[8] Sanyal AJ, Van Natta ML, Clark J, Neuschwander-Tetri BA, Diehl A, Dasarathy S, et al. Prospective study of outcomes in adults with nonalcoholic fatty liver disease. N Engl J Med 2021;385:1559–1569.

[9] Angulo P, Kleiner DE, Dam-Larsen S, Adams LA, Bjornsson ES, Charatcharoenwitthaya P, et al. Liver fibrosis, but no other histologic features, is associated with long-term outcomes of patients with nonalcoholic fatty liver disease. Gastroenterology 2015;149:389–397.e10.

[10] Karsdal MA, Daniels SJ, Holm Nielsen S, Bager C, Rasmussen DGK, Loomba R, et al. Collagen biology and non-invasive biomarkers of liver fibrosis. Liver Int 2020;40:736–750.

[11] Karsdal MA, Krarup H, Sand JMB, Christensen PB, Gerstoft J, Leeming DJ, et al. Review article: the efficacy of biomarkers in chronic fibroproliferative diseases – early diagnosis and prognosis, with liver fibrosis as an exemplar. Aliment Pharmacol Ther 2014;40:233–249.
[12] Daniels SJ, Leeming DJ, Eslam M, Hashem AM, Nielsen MJ, Krag A, et al. ADAPT: an algorithm incorporating PRO-C3 accurately identifies patients with NAFLD and advanced fibrosis. Hepatology 2019;69:1075–1086.
[13] Nielsen MJ, Karsdal MA, Kazankov K, Grønbæk H, Krag A, Leeming DJ, et al. Fibrosis is not just fibrosis – basement membrane modelling and collagen metabolism differs between hepatitis B- and C-induced injury. Aliment Pharmacol Ther 2016;44:1242–1252.
[14] Thiele M, Johansen S, Gudmann NS, Madsen B, Kjærgaard M, Nielsen MJ, et al. Progressive alcohol-related liver fibrosis is characterised by imbalanced collagen formation and degradation. Aliment Pharmacol Ther 2021;54:1070–1080.
[15] Leeming DJ, Karsdal MA, Byrjalsen I, Bendtsen F, Trebicka J, Nielsen MJ, et al. Novel serological neo-epitope markers of extracellular matrix proteins for the detection of portal hypertension. Aliment Pharmacol Ther 2013;38:1086–1096.
[16] Nielsen MJ, Nedergaard AF, Sun S, Veidal SS, Larsen L, Zheng Q, et al. The neo-epitope specific PRO-C3 ELISA measures true formation of type III collagen associated with liver and muscle parameters. Am J Transl Res 2013;5:303–315.
[17] Ryder SD. Progression of hepatic fibrosis in patients with hepatitis C: a prospective repeat liver biopsy study. Gut 2004;53:451–455.
[18] Lawson A, Hagan S, Rye K, Taguri N, Ratib S, Zaitoun AM, et al. The natural history of hepatitis C with severe hepatic fibrosis. J Hepatol 2007;47:37–45.
[19] Maurice JB, Brodkin E, Arnold F, Navaratnam A, Paine H, Khawar S, et al. Validation of the Baveno VI criteria to identify low risk cirrhotic patients not requiring endoscopic surveillance for varices. J Hepatol 2016;65:899–905.
[20] Galle PR, Forner A, Llovet JM, Mazzaferro V, Piscaglia F, Raoul J-L, et al. EASL Clinical Practice Guidelines: management of hepatocellular carcinoma. J Hepatol 2018;69:182–236.
[21] Angeli P, Bernardi M, Villanueva C, Francoz C, Mookerjee RP, Trebicka J, et al. EASL Clinical Practice Guidelines for the management of patients with decompensated cirrhosis. J Hepatol 2018;69:406–460.
[22] Guha IN, Parkes J, Roderick P, Chattopadhyay D, Cross R, Harris S, et al. Noninvasive markers of fibrosis in nonalcoholic fatty liver disease: validating the European Liver Fibrosis Panel and exploring simple markers. Hepatology 2007;47:455–460. https://doi.org/10.1002/hep.21984.
[23] Božin T, Mustapić S, Bokun T, Patrlj L, Rakić M, Aralica G, et al. ALBI score as a predictor of survival in patients with compensated cirrhosis resected for hepatocellular carcinoma: exploratory evaluation in relationship to PALBI and MELD liver function scores. Acta Clin Croat 2018;57:292–300.
[24] Vesterhus M, Hov JR, Holm A, Schrumpf E, Nygård S, Godang K, et al. Enhanced Liver Fibrosis score predicts transplant-free survival in primary sclerosing cholangitis. Hepatology 2015;62:188–197.
[25] Peters MG, Bacchetti P, Boylan R, French AL, Tien PC, Plankey MW, et al. Enhanced liver fibrosis marker as a noninvasive predictor of mortality in HIV/hepatitis C virus-coinfected women from a multicenter study of women with or at risk for HIV. AIDS 2016;30:723–729.
[26] Parkes J, Roderick P, Harris S, Day C, Mutimer D, Collier J, et al. Enhanced liver fibrosis test can predict clinical outcomes in patients with chronic liver disease. Gut 2010;59:1245–1251.
[27] Mayo MJ, Parkes J, Adams-Huet B, Combes B, Mills AS, Markin RS, et al. Prediction of clinical outcomes in primary biliary cirrhosis by serum enhanced liver fibrosis assay. Hepatology 2008;48:1549–1557.
[28] Nielsen MJ, Veidal SS, Karsdal MA, Orsnes-Leeming DJ, Vainer B, Gardner SD, et al. Plasma Pro-C3 (N-terminal type III collagen propeptide) predicts fibrosis progression in patients with chronic hepatitis C. Liver Int 2015;35:429–437.
[29] Karsdal MA, Henriksen K, Nielsen MJ, Byrjalsen I, Leeming DJ, Gardner S, et al. Fibrogenesis assessed by serological type III collagen formation identifies patients with progressive liver fibrosis and responders to a potential antifibrotic therapy. Am J Physiol Gastrointest Liver Physiol 2016;311:G1009–G1017.
[30] Karsdal MA, Hjuler S, Luo Y, Rasmussen DG, Nielsen MJ, Holm Nielsen S, et al. Assessment of liver fibrosis progression and regression by a serological collagen turnover profile. Am J Physiol Gastrointest Liver Physiol 2019;316:G25–G31.
[31] Bril F, Leeming DJ, Karsdal MA, Kalavalapalli S, Barb D, Lai J, et al. Use of plasma fragments of propeptides of Type III, V, and VI procollagen for the detection of liver fibrosis in type 2 diabetes. Diabetes Care 2019;42:1348–1351.
[32] Praktiknjo M, Lehmann J, Nielsen MJ, Schierwagen R, Uschner FE, Meyer C, et al. Acute decompensation boosts hepatic collagen type III deposition and deteriorates experimental and human cirrhosis. Hepatol Commun 2018;2:211–222.
[33] Lehmann J, Praktiknjo M, Nielsen MJ, Schierwagen R, Meyer C, Thomas D, et al. Collagen type IV remodelling gender-specifically predicts mortality in decompensated cirrhosis. Liver Int 2019;39:885–893.
[34] Nielsen MJ, Villesen IF, Gudmann NS, Leeming DJ, Krag A, Karsdal MA, et al. Serum markers of type III and IV procollagen processing predict recurrence of fibrosis in liver transplanted patients. Sci Rep 2019;9:14857.
[35] Laursen TL, Villesen IF, Leeming DJ, Karsdal MA, Sølund C, Tarp B, et al. Altered balance between collagen formation and degradation after successful direct-acting antiviral therapy of chronic hepatitis C. J Viral Hepat 2020;28:236–244.
[36] Fontana RJ, Dienstag JL, Bonkovsky HL, Sterling RK, Naishadham D, Goodman ZD, et al. Serum fibrosis markers are associated with liver disease progression in non-responder patients with chronic hepatitis C. Gut 2010;59:1401–1409.
[37] Everhart JE, Wright EC, Goodman ZD, Dienstag JL, Hoefs JC, Kleiner DE, et al. Prognostic value of Ishak fibrosis stage: findings from the hepatitis C antiviral long-term treatment against cirrhosis trial. Hepatology 2010;51:585–594.
[38] Nielsen MJ, Thorburn D, Leeming DJ, Hov JR, Nygård S, Moum B, et al. Serological markers of extracellular matrix remodeling predict transplant-free survival in primary sclerosing cholangitis. Aliment Pharmacol Ther 2018;48:179–189.
[39] Vesterhus M, Nielsen MJ, Hov JR, Saf F, Manon-Jensen T, Leeming J, et al. Comprehensive assessment of ECM turnover using serum biomarkers establishes PBC as a high-turnover autoimmune liver disease. JHEP Rep 2021;3:100178.
[40] Madsen BS, Thiele M, Detlefsen S, Kjærgaard M, Møller LS, Trebicka J, et al. PRO-C3 and ADAPT algorithm accurately identify patients with advanced fibrosis due to alcohol-related liver disease. Aliment Pharmacol Ther 2021;54:699–708.
[41] Hirschfield GM, Chazouillères O, Drenth JP, Thorburn D, Harrison SA, Landis CS, et al. Effect of NGM282, a FGF19 analogue, in primary sclerosing Cholangitis: a multicentre, randomized, double-Blind, placebo-controlled phase 2 trial. J Hepatol 2019;70:483–493.
[42] Harrison SA, Rossi SJ, Paredes AH, Trotter JF, Bashir MR, Guy CD, et al. NGM282 improves liver fibrosis and histology in 12 weeks in patients with nonalcoholic steatohepatitis. Hepatology 2020;71:1198–1212.
[43] Hartman ML, Sanyal AJ, Loomba R, Wilson JM, Nikooienejad A, Bray R, et al. Effects of novel dual GIP and GLP-1 receptor agonist tirzepatide on biomarkers of nonalcoholic steatohepatitis in patients with type 2 diabetes. Diabetes Care 2020;43:1352–1355.
[44] Sanyal A, Charles ED, Neuschwander-Tetri BA, Loomba R, Harrison SA, Abdelmalek MF, et al. Pegbelfermin (BMS-986036), a PEGylated fibroblast growth factor 21 analogue, in patients with non-alcoholic steatohepatitis: a randomised, double-blind, placebo-controlled, phase 2a trial. Lancet 2019;392:2705–2717.

Journal of Hepatology, Volume *5*

Supplemental information

PRO-C3 is a predictor of clinical outcomes in distinct cohorts of patients with advanced liver disease

Mette J. Nielsen, Grace E. Dolman, Rebecca Harris, Peder Frederiksen, Jane Chalmers, Jane I. Grove, William L. Irving, Morten A. Karsdal, Keyur Patel, Diana Julie Leeming, and Indra Neil Guha

PRO-C3 is a predictor of clinical outcomes in distinct cohorts of patients with advanced liver disease

Mette J Nielsen[1], Grace E Dolman[2], Rebecca Harris[2], Peder Frederiksen[1], Jane Chalmers[2], Jane Grove[2,5], William L Irving[2,4], Morten A Karsdal[1], Keyur Patel[3], Diana Julie Leeming[1], Indra Neil Guha[2,5]

Table of contents

Fig. S1……………………………………………………………………………………1

Fig. S2……………………………………………………………………………………2

Fig. S1

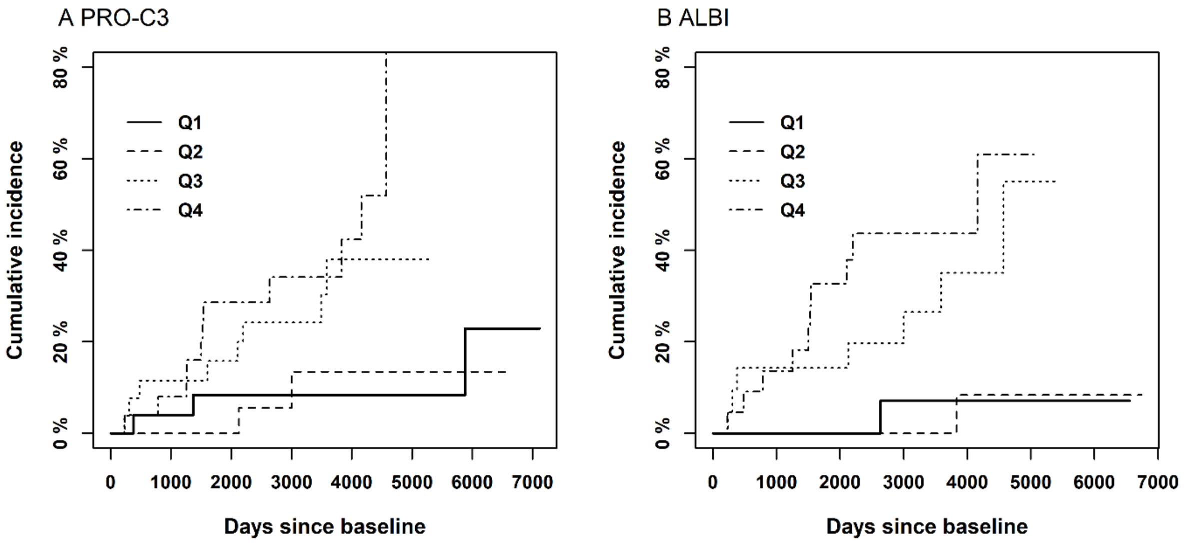
A PRO-C3
B ALBI
Cumulative incidence
0 %
20 %
40 %
60 %
80 %
Q1
Q2
Q3
Q4
0
1000
2000
3000
4000
5000
6000
7000
Days since baseline

Fig. S2

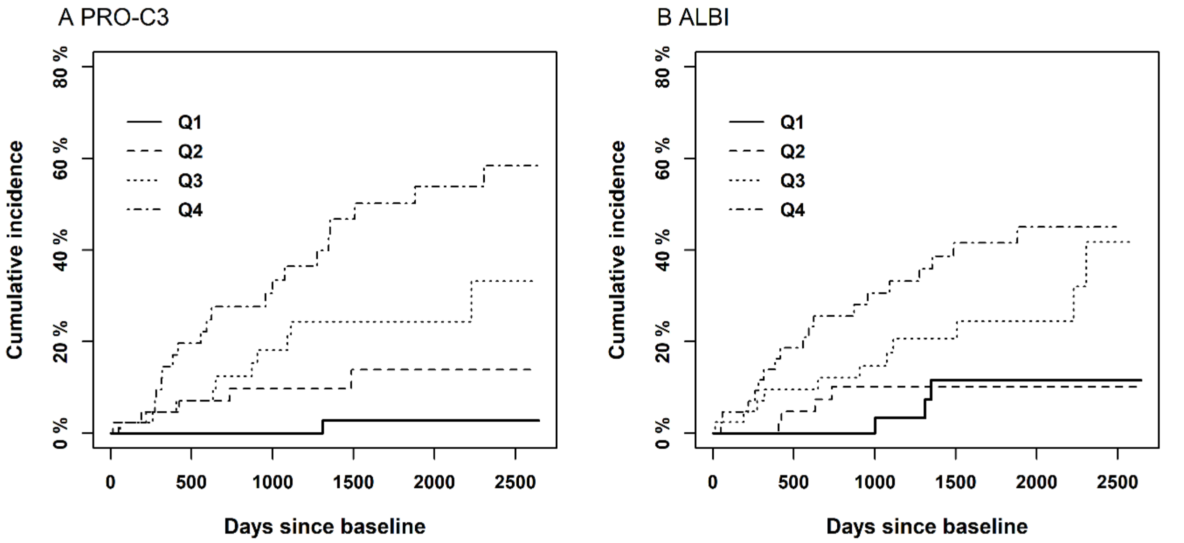
A PRO-C3
B ALBI
Cumulative incidence
0 %
20 %
40 %
60 %
80 %
Q1
Q2
Q3
Q4
0
500
1000
1500
2000
2500
Days since baseline
